# Review of *in silico* studies dedicated to the nuclear receptor family: Therapeutic prospects and toxicological concerns

**DOI:** 10.3389/fendo.2022.986016

**Published:** 2022-09-13

**Authors:** Asma Sellami, Manon Réau, Matthieu Montes, Nathalie Lagarde

**Affiliations:** Laboratoire GBCM, EA 7528, Conservatoire National des Arts et Métiers, Hésam Université, Paris, France

**Keywords:** nuclear receptors, in silico, endocrine disrupting chemicals, docking, pharmacophore model, QSAR

## Abstract

Being in the center of both therapeutic and toxicological concerns, NRs are widely studied for drug discovery application but also to unravel the potential toxicity of environmental compounds such as pesticides, cosmetics or additives. High throughput screening campaigns (HTS) are largely used to detect compounds able to interact with this protein family for both therapeutic and toxicological purposes. These methods lead to a large amount of data requiring the use of computational approaches for a robust and correct analysis and interpretation. The output data can be used to build predictive models to forecast the behavior of new chemicals based on their *in vitro* activities. This atrticle is a review of the studies published in the last decade and dedicated to NR ligands *in silico* prediction for both therapeutic and toxicological purposes. Over 100 articles concerning 14 NR subfamilies were carefully read and analyzed in order to retrieve the most commonly used computational methods to develop predictive models, to retrieve the databases deployed in the model building process and to pinpoint some of the limitations they faced.

## 1 Introduction

Nuclear receptors (NRs) are a large family of transcription factors. They are involved in a wide variety of biological and physiological processes such as growth, metabolism, reproduction, cell proliferation, differentiation, development and homeostasis (1, 2). The NRs superfamily is composed of 48 members in humans and is divided in 7 subgroups. Apart from SHP and DAX, all the NRs share the overall same architecture comprising 5 domains, named A to E, each of which playing a specific role. The E domain is a structurally conserved allosteric signaling region forming the ligand binding domain LBD. This domain includes 12 helices and 4 β‐strands that create a buried hydrophobic ligand‐binding pocket (LBP) able to interact with small molecules ligands. The LBD and in particular the helix H12, called the activation function helix (AF‐H), change conformation upon ligand-binding facilitating the interaction of the LBD with co-activators or co-repressors proteins. The DNA binding domain (C domain) of the activated NR can bind to a specific DNA sequence and recruit co-regulator proteins that either promote or repress the DNA transcription and therefore, specific gene expression (1).

Although the mechanism of action of NRs is very well-adjusted, dysfunctions of their signaling pathways have been linked with diseases such as cancers, diabetes or auto-immune disorders. NRs biology and ability to interact with small lipophilic molecules in the LBD have led to their emergence as a major class of therapeutic drug targets for these diseases, accounting for more than 10% of FDA-approved drugs (1–5).

However, exogenous ligand binding may also cause dysfunctions of NRs pathways. As such, some (candidate) drugs side effects are related to their unforeseen interaction with NRs like nifedipine and the antifungal clotrimazole who are able to activate PXR (6). More recently, a category of compounds called the endocrine disrupting chemicals (EDCs), have been associated with NRs, especially the sex hormones receptors such as Estrogen Receptors (ER) and Androgen Receptors (AR). Although the concept has been introduced since 1958, the scientific outline has evolved to settle in 2012 on a definition proposed by the Endocrine society (7). EDCs are environmental compounds that can be found in fumes, cosmetic additives or pesticides that can enter the human body due to their high lipophilicity (8). They can affect the endocrine system through several mechanisms (8) (9) including “direct” and “indirect” mechanisms. In the “direct” mechanism, EDCs bind to human NRs (hNRs) LBD and dysregulate the functioning of the endocrine system either by excessively activating or repressing the associated biological activity (7, 9). In the “indirect” mechanism, EDCs can alter not only the synthesis, metabolism, transportation and fate of hormones in the body but also the hormone-receptors expression, the signal transduction as well as the epigenic behind (8). Up to 2019, more than 1000 suspected EDCs have been reported (10). It is to note that EDCs can also affect other protein families than the NRs (11–13). For this review, we will focus on the NRs related “direct” mechanism of action.

Identifying hNRs binders, i.e. compounds able to bind to hNRs, is important for both therapeutic and toxicologic purposes: to discover new therapeutic compounds for several NRs-related diseases, to predict potential off-target effects, to guarantee the safety of novel synthesized molecules and to identify potential EDCs in the exposome. *In silico* methods emerge as an asset to achieve this goal. The construction of predictive models of NRs ligand binding using *in silico* methods (14–16) enable to prioritize compounds that should be biologically evaluated to reduce the time, cost and technical issues associated with the experimental tests of a large number of compounds.

Previous reviews mostly focused on specific NRs as potential drug discovery targets (17, 18) while other evaluated models dedicated to EDCs prediction (7, 19). Herein, we present an exhaustive review of 89 *in silico* initiatives carried out on NRs on both therapeutic and toxicologic levels and published between 2010 and 2020. We provide a summary in order to 1) enumerate them in a referential, 2) list the available NRs-related data that can be used in modeling approaches and 3) learn from previous experiences to enable the elaboration of more accurate predictions.

## 2 General overview

This review focuses on 14 NRs subfamily members for which publications falling within the scope of our review were retrieved. 89 articles were carefully selected, read and analyzed. For each collected model, several aspects were identified including 1) the NR for which the model was developed, 2) the computational method used to build the model, 3) the DB used for the model training, testing and validation, 4) the level of reproducibility 5) if the study was prospective or retrospective, and 6) the purpose of the study (toxicological or therapeutic). The result of our bibliographic search is presented in **Table 1**–**4** and point 2) to 4) are detailed in a following paragraph in this review.

**Table 1:** Review of the different initiatives dedicated to Steroid Hormones nuclear receptors.

**Receptor**	**methods**	**Approach**	**Database**	**Reproducibility**	**Prospective or Retrospective**	**Application**	**Year**	**Ref**
**AR**	COMPARA	both	1,746 compounds from ToxCast/Tox21	Medium	retrospective	Toxicological	2020	(20)
**AR**	QSAR : Machine learning methods (kNN, lazy IB1, and ADTree methods)	LB	In house data (292 compounds) and collection from literature (231 compounds)	Medium	prospective	Toxicological	2010	(21)
**AR**	Docking and 3D QSAR (CoMSIA)	both	Collected from the literature (76 compounds)	Medium	retrospective	Toxicological	2013	(22)
**AR**	Docking, MD, and 3D QSAR (Comsia)	both	In house database of flavonoids (21 compounds)	Medium	retrospective	Toxicological	2016	(23)
**AR**	Docking	SB	Collected from the literature (20 bisphenols compounds)	Medium	retrospective	Toxicological	2016	(24)
**AR**	Docking	SB	EPA (1689 compounds)	Medium	prospective	Toxicological	2017	(25)
**AR**	Docking + molecular dynamics	SB	NR-List BDB (3233 compounds)	Medium	retrospective	Toxicological	2018	(26)
**AR**	QSAR : Machine learning methods (Bernoulli Naive Bayes, RF, NNN)	LB	COMPARA calibration set (1689 compounds from EPA) and external validation set (3882 compounds from EPA)	High	retrospective	Toxicological	2019	(27)
**AR**	QSAR : machine learning methods (ANNs,SVM,DT)	LB	CoMPARA dataset (1689 compounds from EPA), EDKB (202 compounds)	Medium	retrospective	Toxicological	2019	(28)
**AR**	Docking and LB Pharmacophore	both	NR-DBIND (812 compounds), Tox21 (5690 compounds)	High	retrospective	Both	2019	(29)
**AR**	QSAR : Machine learning (Bayesian models, RF, kNN,SVM, naïve Bayesian, AdaBoosted DT) and DL	LB	Toxcast (8645 compounds)	High	retrospective	Toxicological	2020	(30)
**AR, ER**	QSAR: KNN + Local method ( lazy learning) + RF	LB	METI (900 compounds), EDKB (87 compounds)	Medium	prospective	Toxicological	2010	(31)
**AR, ER**	QSAR: ML (kNN, DT, NB SVM)	LB	Collected from the literature (1157 compounds in the training set and 121 compounds in the external validation set)	High	retrospective	Toxicological	2014	(14)
**AR, ER**	QSAR: ANN	LB	Collected from the literature (879 compounds for ER, 930 compounds for AR)	High	Retrospective	Toxicological	2015	(32)
**AR, ER**	3D QSAR and bayesian statistics	LB	Toxcast (1853 compounds) + 42 compounds	Medium	retrospective	Toxicological	2016	(33)
**AR, ER alpha**	Hierarchical charactarestic fragments, docking and MD simulations	both	ToxCast/Tox21 and ChEMBL (2458 compounds for ER, 2843 compounds for AR)	Medium	prospective	Toxicological	2020	(34)
**AR, GR**	Similarity	LB	Toxcast (7027 compounds for AR, 7329 compounds for GR)	High	retrospective	Toxicological	2020	(35)
**ER**	CERAPP	both	Collected from the literature (1677 compounds)	Medium	retrospective	Toxicological	2016	(36)
**ER**	QSAR: ANN	LB	Collected from the literature (174 compounds)	Medium	Both	Toxicological	2010	(37)
**ER**	QSAR (single task an multi task learning KNN) and docking	both	Collected from the literature including EDKB and ChEMBL (QSAR data sets: 546 compounds for ERa, 137 compounds for ERb; docking data sets: 106 binders/ 4018 decoys for ERa, 80 binders/ 2000 for ER b)	Medium	Both	Toxicological	2013	(38)
**ER**	QSAR:machine learning methods (LDA / CART/SVM)	LB	Toxcast (1814 compounds) and Tox21 (8303 compounds)	Low	retrospective	Toxicological	2013	(39)
**ER**	Docking	SB	EPA (1677 compounds)	Medium	retrospective	Toxicological	2015	(40)
**ER**	Docking and QSAR :machine learning methods (LDA, decision tree, SVM)	both	Collected from the literature (440 compounds)	Medium	retrospective	Toxicological	2016	(41)
**ER**	QSAR: Machine learning (Bernoulli Naive Bayes, AdaBoost Decision Tree, RF, SVM) and deep learning (DNN) methods	LB	Collected from the literature (1677 compounds from the CERAPP data set, 7351 compounds from Tox21, 3474 compounds for ERa, 2775 compounds for ERb)	High	retrospective	Toxicological	2018	(42)
**ER**	QSAR: Machine learning method (GkNN)	LB	ToxCast and CERAPP databases (1677 compounds)	Low	retrospective	Toxicological	2018	(43)
**ER**	QSAR: Machine learning method (Bayesian models)	LB	"Toxcast2019" and two publications	Medium	Both	Toxicological	2020	(44)
**ER**	QSAR: Machine-learning (BNB, kNN, RF, and SVM) and deep learning (DNN) methods	LB	ToxCast and Tox21 (7576 compounds)	Medium	retrospective	Toxicological	2020	(45)
**ER alpha**	3D QSAR + 2D QSAR : machine learning methods (PLS, SVR, LR)	LB	In house (68 raloxifene's derivatives)	Low	prospective	Therapeutic	2013	(46)
**ER alpha**	Docking	SB	Ligands extracted from cristallographic complexes (66 compounds) and DUD-E's set (106 binders, 4018 decoys)	Medium	retrospective	Therapeutic	2014	(47)
**ER alpha**	Docking and aggregated potential field similarity	both	NCTRER binding database, ChEMBL, DUD (1691 active and 4785 inactive/decoy compounds) and Tox21 for prospective screening	Medium	prospective	Toxicological	2014	(48)
**ER alpha**	Docking	SB	Drug-Bank Database and collection from literature (105 compounds)	Medium	prospective	Therapeutic	2019	(49)
**ER alpha**	QSAR : Machine learning (RF)	LB	EABD (3308 compounds) and Toxcast (1641 compounds)	Medium	retrospective	Toxicological	2015	(50)
**ER alpha and ER beta**	QSAR: ANN	LB	Collected from the literature (170 compounds)	Low	retrospective	Toxicological	2011	(51)
**ER beta**	LB Pharmacophore modeling and QSAR (MLR)	LB	Collected from the literature (119 compounds) and NCI list of compounds for prospective screening	Medium	prospective	Therapeutic	2010	(52)
**ER beta**	LB pharmacophore modeling and docking	both	Maybridge and Enamine	Low	prospective	Therapeutic	2014	(53)
**ER beta**	docking and MD simulations	SB	18 ligands from crystal structures, 40 compounds collected from the literature and 2570 DUD decoys, 400000 compounds from commercial databases for prospective screening	Medium	prospective	Therapeutic	2014	(54)
**ER beta**	QSAR: Machine learning methods (Naïve bayes, KNN, RF, SVM)	LB	CHEMBL20 (356 active compounds and 107 inactive compounds) + 249 DUD-E decoys	Medium	retrospective	Therapeutic	2016	(55)
**ER beta**	QSAR: Machine learning (RF)	LB	EADB (2492 compounds) and ToxCast (1805 compounds)	Medium	retrospective	Both	2017	(56)
**PR**	Docking, MD, Binding energy calculation	SB	Collected from the literature (12 compounds); ZINC db (20000 compounds) for prospective screening	High	prospective	Therapeutic	2018	(57)

**Table 2:** Review of the different initiatives dedicated to RXR and its partners NR.

Receptor	methods	Approach	Database	Reproducibility	Prospective or Retrospective	Application	Year	Ref
**FXR**	SB pharmacophores	SB	ChEMBL (221 compounds); NCI database (247041 compounds) for prospective screening	Medium	prospective	Therapeutic	2011	(58)
**FXR**	SB pharmacophores	SB	in-house Chinese Herbal Medicine database (10216 compounds) for prospective screening	Low	prospective	Therapeutic	2011	(59)
**FXR**	LB Pharmacophore and free energy calculations	LB	ChemBridge (~520000 compounds) for prospective screening	Low	prospective	Therapeutic	2015	(60)
**FXR**	QSAR: Machine learning methods (SVM, C4.5 DT, k-NN, RF, NV), MoSS and SARpy	LB	Tox21 (688 compounds), ChEMBL (460 compounds), D3R CG2 (76 compounds)	Low	retrospective	Toxicological	2018	(61)
**FXR**	QSAR: Machine Learning (counter-propagation artificial neural network, kNN)	LB	ChEMBL (896 compounds), Asinex (3383942 compounds) for prospective screening	Medium	prospective	Therapeutic	2018	(62)
**LXR**	SB pharmacophore and shape similarity	both	Collected from the literature 41 compounds + 67059 decoys from Derwent World Drug Index); NCI database (250761 compounds) for prospective screening	Medium	prospective	Therapeutic	2012	(63)
**LXR**	self-organizing maps (SOM)	LB	ChEMBL (458 compounds); DrugBank (1280 compounds) for prospective screening	Low	prospective	Therapeutic	2017	(64)
**LXR beta**	2D fragment-based HQSAR and HQSSR (structure selectivity) and Docking	both	Collected from the literature (62 quinolines and cinnolines)	Medium	prospective	Therapeutic	2012	(65)
**LXR alpha and LXR beta**	Docking and MD	SB	ChEMBL database + DecoyFinder (769 compounds for LXRa, 570 compounds for LXRb); MolMall subset of the ZINC (~20000 compounds) for prospective screening	High	prospective	Therapeutic	2018	(66)
**LXR beta**	QSAR (MLR) and Docking	both	Collected from the literature (53 compounds with dual activity LRα/β)	Medium	prospective	Therapeutic	2018	(67)
**PPAR alpha and PPAR gamma**	2D-, 3D-QSAR and docking	both	In-house library (22 compounds)	Medium	prospective	Therapeutic	2013	(68)
**PPAR alpha and PPAR gamma**	QSAR, SB pharmacophore modelling and docking	both	In-house library (46 phenylpropanoic acid derivatives)	Medium	prospective	Therapeutic	2016	(69)
**PPAR alpha and PPAR gamma**	docking and MD	SB	Asinex (292,724 compounds) for prospective screening	Medium	prospective	Therapeutic	2018	(70)
**PPAR alpha and PPAR gamma**	docking, binding energy calculations, MD	SB	ChemDiv database (7476 compounds) for prospective screening	Low	prospective	Therapeutic	2019	(71)
**PPAR alpha and PPAR gamma**	Docking and MD	SB	Ligand Expo components database	Medium	prospective	Therapeutic	2020	(72)
**PPAR gamma**	LB Pharmacophores and 3D QSAR	LB	Collected from the library (88 compounds)	Medium	retrospective	Therapeutic	2010	(73)
**PPAR gamma**	QSAR :Machine learning methods (MLR, SVM and Bayes Network Toolbox (BNT)), docking and MD	both	Traditional Chinease Medicine (TCM) database (9,029 compounds)	High	prospective	Therapeutic	2014	(74)
**PPAR alpha and PPAR gamma**	Docking and MD	SB	Compounds collected from the literature (51 compounds + 3600 DUD decoys); "clean-leads" ZINC's subset for prospective screening (740000 compounds)	Low	prospective	Therapeutic	2015	(75)
**PPAR gamma**	SB and LB pharmacophore-, shape similarity and docking	both	Collected from the literature (51 partial agonists, 14 agonists + 812 inactives from ToxCast and literature); Maybridge database (52000 compounds) for prospective screening	Low	prospective	Therapeutic	2016	(76)
**PPAR gamma**	docking and MD	SB	Zbc subset of ZINC database (180313 compounds)	Low	prospective	Therapeutic	2018	(77)
**PPAR gamma**	Docking, binding energy calculations and MD simulations	SB	Seaweed Metabolite Database (1110 compounds)	Medium	prospective	Therapeutic	2021	(78)
**CAR**	docking,SB and LB pharmacophore, QSAR (SVM)	both	Collected from the literature (392 compounds)	Low	retrospective	Therapeutic	2017	(79)
**CAR, PXR**	Docking	SB	Collected from the literature (106 compounds)	Medium	retrospective	Toxicological	2017	(80)
**PXR**	Docking and QSAR (Bayesian classification)	both	Toxcast (308 compounds)	medium	prospective	Toxicological	2010	(81)
**PXR**	QSAR : C5.0	LB	In-house collection (202 compounds) and collection from the literature (434 compounds)	High	retrospective	Both	2012	(82)
**PXR**	QSAR: partial logistic regression(PLR)	LB	Collected from the literature (631 compounds)	medium	retrospective	Both* (PXR activation is an unwanted side effects of drugs)	2012	(83)
**PXR**	QSAR, similarity	LB	Prestwick Chemical Library (1120 compounds)	Low	prospective	Both* (PXR activation is an unwanted side effects of drugs)	2015	(84)
**PXR**	SB Pharmacophore and docking	SB	Binding DB (266 compounds); PubChem (820 herbs compounds) for prospective screening	Medium	prospective	Both* (PXR activation is an unwanted side effects of drugs)	2015	(85)
**PXR**	SB Pharmacophore	SB	Collected from the literature (18 compounds), Mitsubishi Tanabe Pharma Corporation (68 compounds), NPC (2816 compounds)	Low	retrospective	Both* (PXR activation is an unwanted side effects of drugs)	2017	(86)
**TR**	Docking and MD simulations	both	Collected from the literature (16 HO-PBDEs compounds)	Medium	retrospective	Toxicological	2016	(87)
**TR**	QSAR (C4.5 ,SVM and Random Forest)	LB	Collected from the literature (258 compounds)	Medium	retrospective	Toxicological	2019	(88)
**TR beta**	Docking and MD	SB	DUD-E (7556 compounds), in-house indoor dust contaminant inventory (485 compounds)	Medium	retrospective	Toxicological	2016	(89)
**TR beta**	3D QSAR, Docking and MD	both	Collected from the literature (33 compounds)	Medium	retrospective	Therapeutic	2015	(90)
**TR beta**	Docking and QSAR (PLS)	both	Collected from the literature (18 HO-PBDEs compounds)	Medium	prospective	Toxicological	2010	(91)
**VDR**	de novo design, docking, MD, free energy calculation	both	Fragments extracted from 6 VDR agonists collected from the literature	Low	prospective	Therapeutic	2012	(92)
**VDR**	Docking, LB Pharmacophores, 3D QSAR, MD	both	ChEMBL (478 compounds)	Medium	retrospective	Therapeutic	2020	(93)
**VDR**	LB pharmacophore, molecular docking, binding free energy calculation, Density Functional Theory (DFT) study and MD	both	Binding database (31 compounds); for prospective screening: Life chemicals, Enamine, MayBridge, and TCM	Low	prospective	Therapeutic	2020	(94)

**Table 3:** Review of the different initiatives dedicated to monomeric orphan receptors.

Receptor	methods	Approach	Database	Reproducibility	Prospective or Retrospective	Application	Year	Ref
**ERR**	Combination of QSAR models	LB	Tox21 (5077 compounds for ERR agonism, 6526 compounds for ERR inhibition); HMDB (3092 compounds) and EU pesticides dataset (888 compounds) for prospective screening	high	prospective	Toxicological	2019	(95)
**ERR**	molecular similarity and docking	both	KEGG COMPOUND database (10739 compounds)	high	prospective	Therapeutic	2013	(96)
**LRH-1**	Docking	SB	ZINC database (5.2 million compounds) for prospective screening	Low	prospective	Therapeutic	2013	(97)
**RORγt**	docking and similarity	both	ChEMBL (502 compounds); Specs commercial database (116495 compounds) for prospective screening	Medium	prospective	Therapeutic	2018	(98)
**RORγt**	SB pharmacophore and Docking	SB	Asinex Gold–Platinum (289174 compounds)	Medium	prospective	Therapeutic	2020	(99)

**Table 4:** Review of the different initiatives dedicated to projects targeting several NR.

Receptor	methods	Approach	Database	Reproducibility	Prospective or Retrospective	Application	Year	Ref
**AhR, AR, CAR, ER, ERR, FXR, GR,PPARd, PPARg, PR, PXR, RAR, ROR, RXR, TR, VDR**	QSAR : Deep learning method (molecular image-based method)	LB	Tox21 Data Challenge 2014 (~7000 compounds / NRs)	Low	prospective	Toxicological	2020	(100)
**AhR, AR, ER, PPARg**	QSAR: Machine learning (RF) and Deep Learning (Deep Neural Network) methods	LB	Tox21 (10255 compounds curated from the original 12707 compounds)	Medium	retrospective	Toxicological	2016	(101)
**AhR, AR, ER, PPARg**	QSAR :Deep learning method (DNN)	LB	Tox21 (8694 compounds curated from the original 12707 compounds)	Medium	retrospective	Toxicological	2016	(102)
**AhR, AR, ER, PPARg**	QSAR: Machine learning (RF and SVM)/Molecular similarity/ SB Pharmacophore modeling	LB	Tox21 (~7000 compounds / NRs)	High	retrospective	Toxicological	2018	(103)
**AR, ER alpha, ER beta, GR, PPAR alpha, PPAR beta, PPAR gamma, PR, RXR alpha, and TR alpha and TR Beta**	Docking	SB	DUDE-E, ChEMBL	High	retrospective	Toxicological	2014	(104)
**AR, ER,GR, PPAR gamma, TR**	QSAR : Machine learning methods (SVM, RF)	LB	Tox21 (7248 compounds)	High	retrospective	Toxicological	2019	(19)
**AhR, AR, ERα, ERβ, GR, LXR, MR, PPAR gamma, PR, TR alpha, TR beta**	Virtual Tox Lab software (docking and mQSAR)	both	Collected from the literature (1016 compounds)	Medium	prospective	Toxicological	2012 and 2014	(12, 105)
**AR, GR, MR, PPAR alpha, PPAR beta, PPAR gamma, PR, RAR alpha, RXR alpha, TR beta, VDR**	Docking	SB	Collected from the literature (157 compounds)	High	retrospective	Therapeutic	2010	(106)

**Figure 1:**
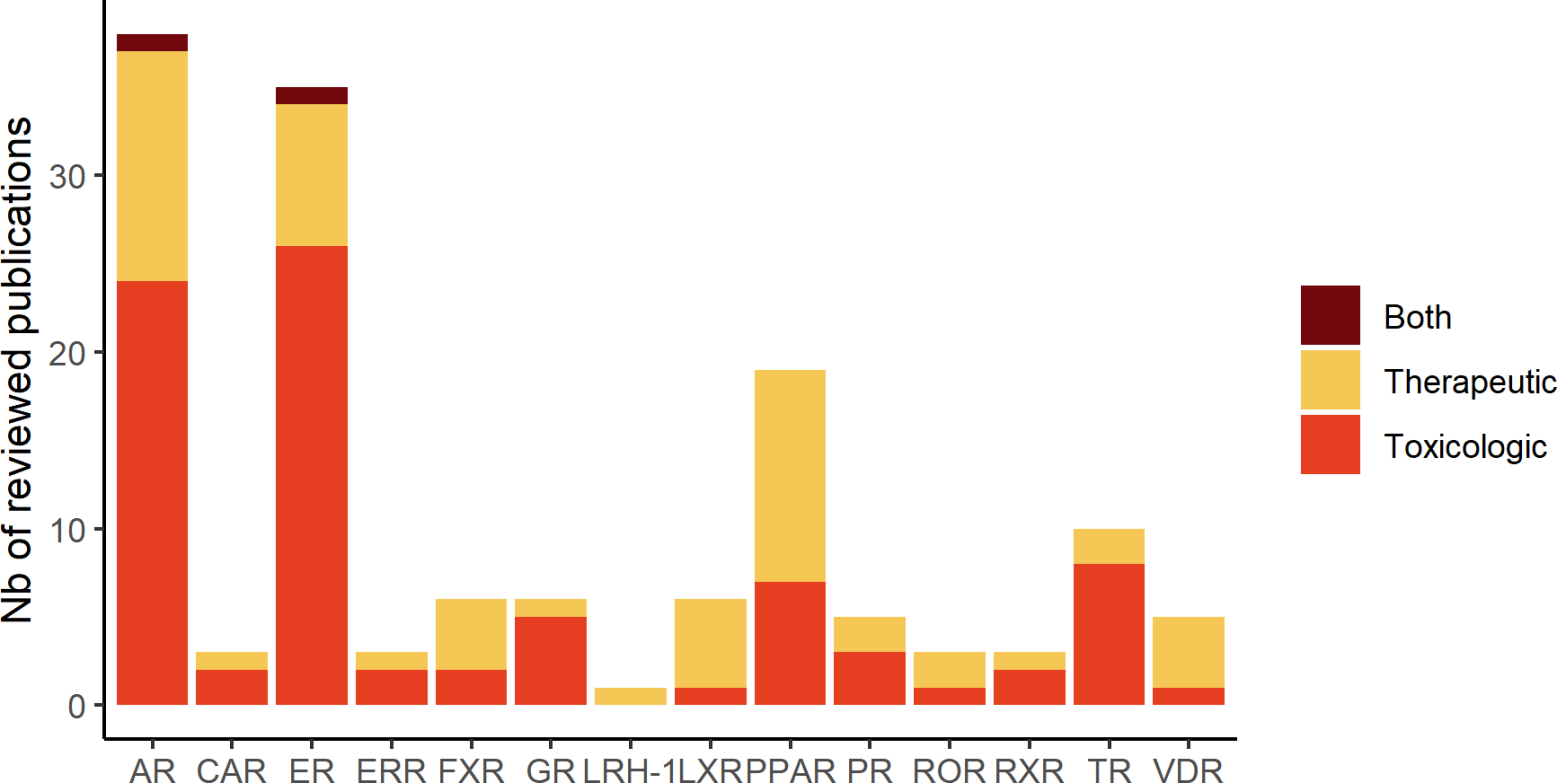
Number of publications related to each studied hNR subfamily described in the review.

**Figure 2:**
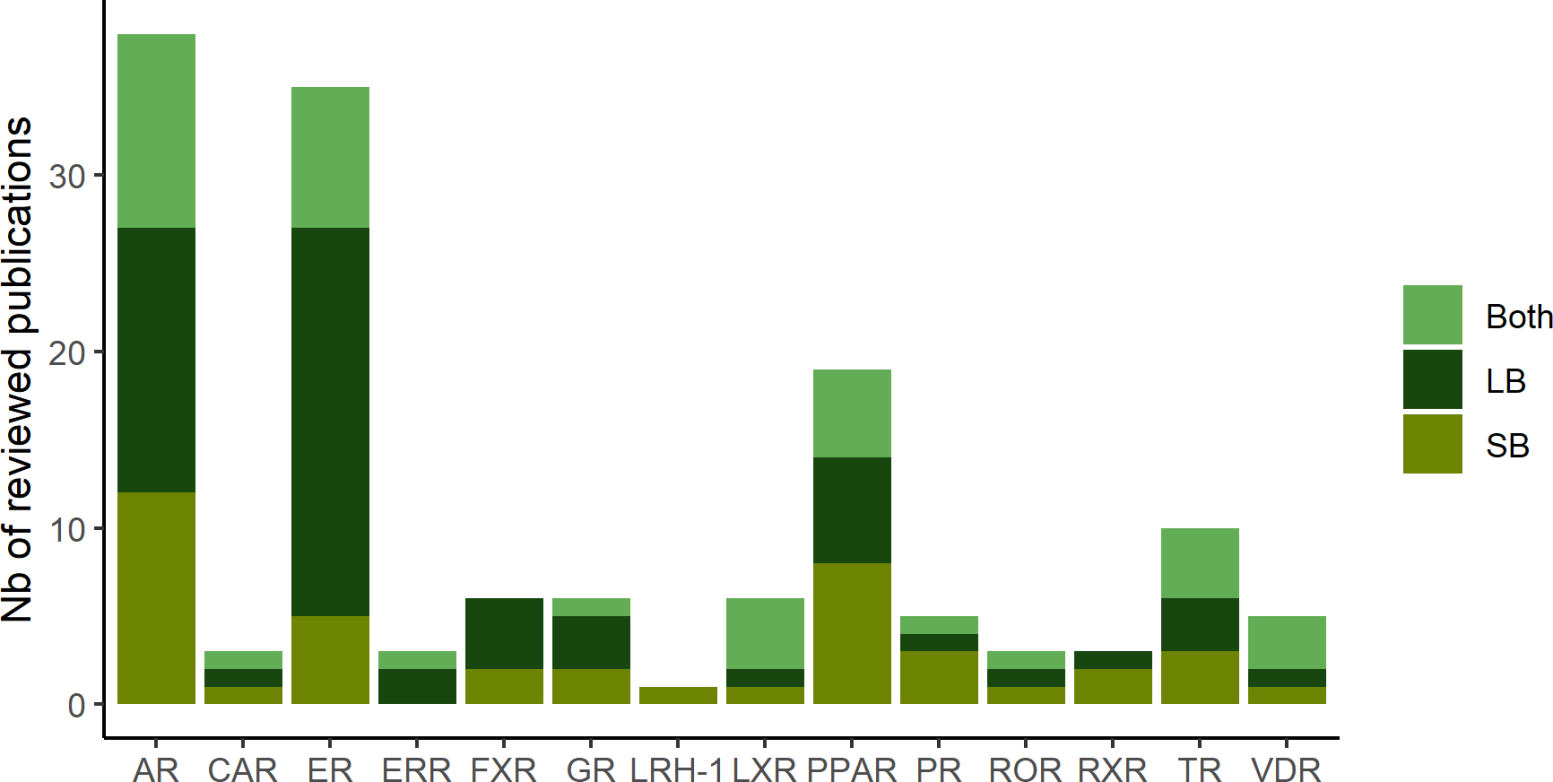
Distribution of the computational approach (SB: structure-based, LB: ligand-based and both: combination of SB and LB methods) in the reviewed publications for the different hNR.

In total, in this review, we identified 38 projects dedicated to the identification of therapeutic compounds, 44 to the prediction of toxicological compounds and 3 projects addressing both purposes. Distribution of publications related to each studied NR are depicted in **Figure 1**. The in silico methods used to construct the models are divided into two approaches: Ligand-Based (LB) and Structure-Based (SB) (**Figure 2**). The reviewed studies are also classified according to their study design into prospective and retrospective studies. Prospective studies are based on compounds for which no biological data is available for the query target. These compounds are subjected to virtual screening protocols in order to assess if they can be considered as potential NRs modulators. The obtained predictions are then validated by experimental assays. In retrospective studies, models that are developed aim at correctly forecasting the already known data activities. The models can then be used to achieve predictions for compounds with unknown activities as long as they remain within the activity domain. The reviewed papers were perfectly balanced in terms of study design as 43 model were retrospective and 43 were prospective with the latter including a majority of LB methods.

## 3 Studied nuclear receptors

In addition to the IUPAC classification, NR can be distinguished according to structural differences, functions, tissue specificity, DNA binding motifs or the knowledge or not about an endogenous ligand. For this review, we divided the studied NR into three groups according to their structural similarities and function in three groups. 1) steroid hormones receptors, (**Table 1**) 2) RXR and its partners (**Table 2**) 3) monomeric orphan receptors (**Table 3**) (107, 108).

### 3.1 Steroid hormones receptors

The NR belonging to this class are all activated by an endogenous ligand presenting a steroid core. Upon binding to their native ligand, steroid hormones receptors undergo conformational changes leading to their homodimerization and subsequently to DNA binding. The reviewed articles dedicated to the steroid hormone receptors are listed in **Table 1**.

#### 3.1.1 Estrogen receptors

Two isoforms of ER exist, namely ERα and ERβ. Both isoforms exhibit similar affinity for their native ligand, 17β Estradiol, but differential expression in the body and unique roles in estrogens action *in vivo* (55). Indeed, ERα’s effects are prominent in the mammary gland, uterus and in the preservation of skeletal homeostasis and the regulation of metabolism, while the activation of ERβ impacts the immune and central nervous systems. Moreover, ERβ exerts an anti-proliferative and pro-apoptotic activity that counteracts ERα’s actions towards cell growth and proliferation (54, 55). The established link between impairment of these ER pathways and diseases such as breast and endometrial cancers, osteoporosis, metabolic and cardiovascular diseases (55, 109) explains the number of published *in silico* studies with a therapeutic scope (8 up to the 23 ER-related reviewed studies). Additionally, ER are a well-known target for EDCs, and it has been shown that exposure to these chemicals increase the risk of breast cancer and immune diseases development (18). Consequently, several projects focused on this hNR as a central component of toxicological pathways including EDCs. It is the case of CERAPP, a large-scale screening project initiated by the US Environmental Protection Agency (EPA) (36). The project gathers models from American as well as European research groups to develop *in silico* models to evaluate thousands of chemicals for ER-related activity and prioritize them for further testing. CERAPP models, like most of the ER focused models reported in the literature, do not differentiate the two ER isoforms during the development of the models. It is to note that this distinction is crucial for therapeutic projects as many ligands display different affinities for each isoform (110). Designing selective ERβ ligands could help reducing ER related side effects besides exerting the desired estrogenic activity. Niu et al. (55), succeeded in building machine leaning models with high accuracies (77.10 to 88.34%) and AUCs greater than 0.8 that performed equally on an external validation dataset composed of ERβ selective agonists. It is to note that the negative data used for this study consisted of generated decoys rather than selective ERα ligands. Another particularity we could notice for ER models, was the predominance of Quantitative Structure Activity Relationship (QSAR) models based on machine learning (ML) algorithms. The most used ML algorithms were random forest, SVM and Naïve Bayes. Other computational approaches comprise molecular docking and pharmacophore modelling.

#### 3.1.2 Androgen receptors

Modulated by dihydrotestosterone and testosterone, AR is involved in several physiological processes such as the male sexual differentiation, the development and maintenance of musculoskeletal and cardiovascular systems, as well as functionality of female ovarian follicles and ovulation (29, 111). The malfunctioning of these receptors is linked to several diseases including prostate, testicular and ovarian cancers, impaired reproduction system development and neuromuscular diseases (29, 112). Despite this large therapeutic potential, the 23 models and projects identified for AR during this review process were for a large majority conducted in the EDCs risk assessment perspective. Among them, the CoMPARA project (20) is the AR counterpart of the CERAPP project and falls under the Endocrine Disruptor Screening Program of the U.S. Environmental Protection Agency (EPA). The 25 international research teams forming the CoMPARA consortium used a common training set of 1,746 chemicals compiled from a data set of 11 ToxCast/Tox21 HTS *in vitro* assays to generate 91 predictive QSAR models for AR binding, agonist, and antagonist activity predictions. The resulting models were evaluated using non-overlapping curated literature data. Finally, all predictions were combined into consensus models with an average accuracy of 80%. In complement to the CoMPARA project, several computational LB models, obtained using deep learning and QSAR methods, were described in the literature. SB methods such as docking and MD were also used, albeit to a lesser extend because of a lack of AR structural data. In fact, there is no AR antagonist-bound crystal structure yet available in the PDB which can impair the prediction of AR antagonist molecules and the development of AR SB models. However, some groups managed to overcome this issue and relied on molecular dynamics simulations to generate AR structures in antagonist conformations that led to a vast improvement for the docking of AR antagonists (26).

#### 3.1.3 Progesterone receptor

PR is expressed primarily in female reproductive tissues and in the central nervous system as two isomers, PRα and PRβ. Both isomers present identical structures except for the A/B domain but they target distinct gene networks in progesterone-responsive cells (113). PR is activated by binding its endogenous ligand, the progesterone. PR regulates a wide range of biological function in a context- and cell-specific manner including the development and differentiation processes in normal and malignant female tissues, in particular in patients suffering from breast cancer (114). Selective progesterone modulators (SPRMs) have thus been developed as drug candidates for breast cancer therapies (57). Moreover, PR is suspected to be involved in endocrine disruptor toxicity pathway as bisphenols and some pesticides have been characterized with a PR activity (107). The reviewed models reflect the involvement of this NRs in both aspects. Three *in silico* endocrine disruptors prediction tools (12, 100, 104, 105) include PR models. In particular, a prediction model of PR agonist compounds was constructed using a deep learning approach (100) and the Tox21 data. This model was associated with high prediction performance as measured by a Matthew correlation coefficient superior to 0.8 and an AUC value of 0.95. A structure-based prospective virtual screening to identify new PR inhibitors was also conducted (57) but no experimental validation of the results was provided.

#### 3.1.4 Glucocorticoid receptors

Besides their well-known anti-inflammatory, anti-proliferative, pro-apoptotic and anti-angiogenic roles, glucocorticoids are involved in several other physiological processes affecting the nervous, cardiovascular, immune and respiratory systems (115, 116). Additionally, GR is incriminated in several toxicological pathways resulting in decreased male fertility and depression (35) and are a potential target for EDCs (19). GR is thus a relevant target for both therapeutic and toxicologic compounds. There are several isoforms of GR distributed through various tissues. GRα and GRβ present a similar sequence that differs only in the C-terminal region, whereas GRγ, GR-A, and GR-P are less characterized. It has been shown that GRβ negatively regulate the action of GRα as well as exerting its own. The remaining isoforms are associated to glucocorticoids insensitivity (115). In this review, only one therapeutic project was identified (106) whereas, 4 projects with toxicological applications for GR were found. Both SB, LB and their combination were applied to identify GR ligands. None of these projects did the distinction between all isoforms despite the difference in affinities towards glucocorticoids (115). It is to note that all the available GR structures in the PDB include the same mutation in helix 5 from a Phenylalanine residue to a Serine to overcome solubility problems during the crystallization experiments (117). However, this mutation is not located in the ligand binding site.

### 3.2 RXR and its partners

RXRα presents the particularity of forming heterodimers with one third of the 48 human NR (107) and is thus able to regulate a wide range of biological functions in a cell and tissue-specific manner (118). This class of NR can be subdivided in permissive heterodimers that can be activated by ligands of either RXR or its partner in the dimer (PPARs, LXRs, FXR, PXR and CAR) and non permissive heterodimers that generally require the RXR to be unbound in order to be activated by the native ligand of the dimer partner (TRs, RARs) (107). The reviewed articles focusing on RXR and its partners are presented in **Table 2**.

#### 3.2.1 Retinoid X receptor alpha

As previously mentioned, RXRα can form heterodimers with several NR partners, but it can also be assembled and activated as an homodimer (108). A synthetical RXRα ligand, bexarotene, is notably marketed for the treatment of cutaneous T cell lymphoma (119). RXRα ligands have also demonstrated neuroprotective properties and are considered for the design of drug candidates for Alzheimer disease treatment (120). No study specifically dedicated to RXRα ligands prediction was identified during this review. However, 3 articles presenting models for several NRs including RXRα were available. Among them, two studies used SB methods. However, one of this study (106) is more focused on the comparison the performance of single and ensemble docking approaches with an active RXR data set limited to 11 compounds. In the other SB study, the RXR DUD-E data set (121) was used with a single docking approach to select the RXRα structure. Overall good performance in predicting RXR ligands was obtained with an AUC value close to 0.8 and an early enrichment factor value EF1% of 8.6. The third study presents a RXRα agonist ligands prediction model developed using a LB deep learning approach (100). The model was trained on the Tox21 database and was associated with an AUC value similar to those obtained in the previous study with the docking approach.

#### 3.2.2 Peroxisome proliferator-activated receptors

Since the cloning of the first PPAR from the rat liver in 1990, the subfamily of PPAR has been enlarged and currently counts three members PPARα, PPARδ and PPARγ. Each isoform is differentially expressed in tissues and associated with different biological functions (122): PPARα is mainly expressed in the liver and regulates lipid metabolism (123); PPARγ is mainly expressed in adipose tissues and controls adipogenesis and carbohydrate metabolism (124); PPARδ is ubiquitously expressed and involved in atherosclerosis, pathologies of the nervous system, embryonic, organ and tissue development and metabolism of lipid and glucose (122). PPARδ is the less studied receptor of this group (75) while PPARα and PPARγ have been extensively investigated as therapeutic targets especially in metabolic diseases like type 2 diabetes (T2D) (68, 71, 78, 125, 126). Recently, PPARγ has also been proposed as a therapeutic target for ovarian cancer (77) and Alzheimer Disease (AD) (78). In this review, 11 models and projects dedicated to PPARs have been identified. The implication of this receptor in several pathologies explains the fact that all project specific to PPAR fall under the therapeutic application. Indeed, 8 studies described prospective virtual screening protocols combining several in *silico* methods for the prediction of dual PPARα/γ agonist ligands with therapeutic applications for T2D (70–72) and of PPARγ ligands (74–78). However, experimental validation was achieved for only 3 of these studies (75–77), which limits the evaluation of the protocols performance. The protocol presented by Kaserer et al. for PPARγ partial agonist ligands prediction (76) is of particular interest since a retrospective study was first achieved to select optimal models for the prospective screening. This protocol combines 3 pharmacophore models (associated with EF and AUC values of 6.5 and 0.92 respectively in the retrospective evaluation), 5 shape-based models (associated with EF and AUC values of 11.0 and 0.83 respectively in the retrospective evaluation), and a docking protocol (associated with EF and AUC values of 2.2 and 0.65 respectively in the retrospective evaluation). The top-ten ranked compounds by the three methods (29 compounds) were biologically evaluated and 9 novel PPARγ ligands were identified. Retrospective studies were also conducted on the PPARα/γ using 2D, 3D-QSAR, and docking (68) and to understand the structural factors responsible for PPARγ agonistic activity using a combined pharmacophoric/3D-QSAR approach (75). As PPARγ data is available from the Tox21 program, several studies trying to model the Tox21 data for a large panel of NRs also present PPARγ dedicated models (19, 100–103). All of these models were associated with high predictive power with AUC values comprised between 0.8 and 0.9.

#### 3.2.3 Human pregnane X receptor

Due to its flexibility and the relative bulkiness of its binding site in comparison with other NRs (80), PXR activity can be modulated by binding to a wide range of endogenous compounds, from bile acids to steroid hormones but also xenobiotics, such as drugs or environmental chemicals (pesticides, phenols, cosmetics, phytoestrogens) that can dysregulate normal physiological functions (127–129). PXR is responsible for the modulation of the expression of enzymes and transporters associated with the metabolism and transport of several drugs. Thus, PXR can mediate drug-drug interactions either by reducing the therapeutic efficacy or by increasing the concentration of reactive metabolites leading to toxicity (86). PXR may also lead to the so called “cocktail effect” that is the adverse effects caused by several chemicals present at the same time exhibiting low individual toxicities (83, 129). In this sense, it is important to identify compounds that activate PXR to avoid such effect and modify the drug design during early stages. Several projects focused on developing models able to identify PXR agonists: Torimoto-Katori et al. (86), designed a pharmacophore with high accuracies (over 0.7) yet with lower sensitivities suggesting that reinforcing the methods with other *in silico* methods could help achieve better performances. This was done by Cui et al. (85), who combined pharmacophore and docking method to detect ingredients from herbs able to activate PXR in order to avoid herb-drug effects. Pharmacophore models achieved similar performances as the model described before with sensibilities equal to 0.54 and specificities of 0.8. Interestingly, adding docking methods on top of pharmacophore models enhanced the performances to a detection rate of 0.6 i.e. the ratio of positive hits among all the database compounds. Three other groups built QSAR models to identify potential activators of PXR. The first one (84), achieved performances of R² = 0.64 and was applied to identify 16 novel activators of PXR. Dybdahl et al. (83), built a model associated with a sensitivity of 82% and a specificity of 85% that was used to identify potential PXR activators in a database of environmental chemicals. Interestingly, these molecules were also linked to cause adverse effects such genotoxicity or teratogenicity for examples.

#### 3.2.4 Liver X receptor

LXR presents two isoforms (LXRα and LXRβ) that are both ubiquitously expressed (65, 67, 130–132). LXR serves primarily as a reverse cholesterol transporter within the lipid metabolism, protecting cells from cholesterol excess (66, 67) and is involved in many physiological processes. Thus, LXR represents a promising therapeutic target for cardiovascular diseases, dyslipidemia and cancer treatment (66). However, it has been shown that LXRα activation can lead to undesired lipogenic effects like increased hepatic lipogenesis or liver steatosis whereas LXRβ activation does not and can even reduce them (67). Designing LXRβ selective compounds to treat dyslipidemia appears as a promising strategy yet difficult to achieve due to the high similarity between both isoforms LBD. For this reason, only two projects among the reviewed ones were dedicated to the identification of LXRβ selective agonists whereas the remaining two others did not make any distinction. Both projects used a virtual screening workflow based on a combination of LB and SB methods. In the first article, Chen et al. (67) built a selective LB model using an association of Kohonen maps and stepwise multiple regression. The model relied on the structures of newly reported dual agonists and was associated with *R* ^2^ equal to 0.837 and 0.843 for train and test set respectively. This model was then used to perform predictions of potential selective ligands from the ZINC database falling within the applicability domain of the model. A promising compound was found with a predicted pEC50 = 7.0 for LXRβ and pEC50 = 6.095 for LXRα and was used as template to design potential inhibitors. The latter molecules were incorporated to a docking analysis to better understand the underlying mechanism of the selective activity. Similarly, the second article (65) was also a combination of LB (QSAR) and SB (docking) methods to firstly unravel new selective LXRβ ligands and then analyze the interaction mode using docking studies. No toxicological study related to LXR was collected during our bibliographic search.

#### 3.2.5 Constitutive androstane receptor

CAR is involved in regulation of the transcription of genes encoding for the metabolism of xenobiotic and steroid (133). It is highly expressed in the liver and to less extend the small intestine. Although structurally similar to other NR, the LBD of CAR contains particular residues and motifs and thus present an original conformation. The CAR LBD in its unbound form present a similar conformation to those of other NR when bound to their endogenous ligands. Thus, in the absence of ligand CAR can recruit coactivators. CAR agonist compounds, referred to as “phenobarbital alike”, can increase coactivator recruitment and thus promote the expression of cytochrome P450 enzyme and other proteins involved in metabolism of xenobiotic compounds. Besides the agonist compounds, inverse agonists, such as the androstane metabolites, are also able to bind to CAR LBD. Upon binding to CAR, these compounds inhibit the CAR constitutive activity through the release of coactivators (134, 135). CAR is considered to be a sensor to several xenobiotics including EDCs such as phthalates and triclocarban but it is also considered as an interesting therapeutic target for metabolic diseases such as type 2 diabetes (79, 80). Only few attempts to develop *in silico* models for CAR were found (79, 80, 100). One therapeutic study was conducted with the dual objective of predicting CAR agonist compounds and collecting knowledge about CAR/ligand interactions. To do so, Lee et al. (79) developed a machine-learning model based on pharmacophoric descriptors with good predictive power with accuracy values equal to 0.875 and 0.854 for the training and test sets, respectively and MCCs values equal to 0.744 and 0.701 for the training and test sets, respectively. Additionally, the group identified the critical elements involved in the binding affinity with CAR. Additionally, two toxicological studies that aim to understand the effect of EDCs on the CAR were also retrieved.

#### 3.2.6 Farnesoid X receptor

FXR is expressed in the liver, the kidney, the intestine, and the adrenal glands. It regulates the metabolism of glucose and lipids and the maintenance of the bile acids homeostasis. It is thus a suitable therapeutic target for the prevention and treatment of metabolic syndrome, dyslipidemia, atherosclerosis, and type 2 diabetes (59). Additionally, some exogenous compounds able to bind to FXR can induce the dysfunction of the receptor and are suspected to be responsible for liver toxicity and hepato-biliary injuries (61). In this review, 4 *in silico* models dedicated to the identification of FXR modulators for the treatment of hepatic and metabolic disorders have been analyzed. As a limited number of diverse known FXR modulators was available, SB methods used to be elected to identify potential therapeutic compounds. 3 studies present the generation of FXR SB pharmacophore models and their use in prospective screening in combination with experimental testing to identify FXR agonist hits (57, 59, 60). For example, in the study of Schuster et al. (58), a set of SB pharmacophore models was generated and theoretically evaluated by calculating the enrichment factors using several data sets. The combination of all pharmacophore models was able to retrieve 87.8% of a list of FXR actives but the performance obtained varied according to the model and to the data set studied. EF values were used to produce pharmacophore models ranking for each data set and the 3 most suitable pharmacophore models were selected accordingly for prospective screening. In complement, 3 LB approaches were described in the literature. The Tox21 FXR agonism and antagonism assay data were used in 2 different studies to obtain respectively FXR disruptors model using machine learning methods (61) and separated FXR agonism and FXR antagonism models using a deep learning approach (100). Both studies present high predictive accuracy with area under the ROC curve values reaching 0.8. The third study focus on different machine learning methods (counter-propagation artificial neural network, similarity of 3D pharmacophore feature distributions method and k-nearest neighbor learner) that were optimized and combined to identify new FXR modulators molecular frameworks (62). This ensemble machine learning approach was used in a prospective screening of 3 million commercially available compounds and enable the discovery of 4 new experimentally validated FXR agonist and 2 FXR antagonist compounds with original molecular frameworks.

#### 3.2.7 Thyroid hormones receptors

TR, are regulated endogenously by the thyroid hormones (TH) that play major role in metabolism and growth processes (136). The 2 isoforms, TRα and TRβ, are expressed differently in tissues and play different roles in the TH signaling (137). Despite the great potency of TR as therapeutic target in the field of dyslipidemia and liver diseases, the development of TR modulators has been limited by selectivity problems and associated undesirable side effects on heart and bone. TRα activation being associated with the cardiac effects of TH, TRβ selective compounds are currently investigated for the treatment of metabolic and brain disorders (138). Several environmental chemicals have also been documented as TR modulators, some of them being suspected to be EDCs. It is notably the case of Hydroxylated polybrominated diphenyl ethers that present structural similarity with endogenous TH and may interfere with the TH binding to TR (87). TR are thus largely studied in the EDCs context (139, 140). In this review, 10 models dedicated to TR are listed, including 3 models specifically developed for TRβ and 4 tools to predict potential EDCs that include SB (12, 104, 105) and LB (19, 100) models for a panel of NRs and not only TR. It is to note that these LB initiatives trained their model using the TR Tox21 dataset and that the associated performance was among the lowest of all studied receptors. Among the TR focused studies, Zhang et al. (89) presented a virtual screening protocol combining ensemble docking and MM-GBSA rescoring to identify TRβ ligands. This protocol was developed and evaluated using the TRβ DUD-E data set and was associated with an AUC value of 0.865 and an EF10% value of 7.418. This protocol was then applied in a prospective virtual screening of an indoor dust contaminants inventory but as it was developed using the TRβ DUD-E dataset, it could also be applied to identify potential TRβ binders for therapeutic applications. Among the remaining studies, it is to note that one was dedicated to the classification of TR agonist and antagonist compounds using ML methods but was not applicable to the classification of TR binders and TR non binders (88), and 2 studies using QSAR models were associated with good predictive performance of TRβ agonist compounds (90) and TRβ binders (91) respectively, but both suffer from a limited (<30) number of compounds included in the training and test sets.

#### 3.2.8 Vitamin D receptor

VDR is expressed in various tissues especially in the gastrointestinal tract and the kidneys. It plays a major role in the regulation of vitamin D thus controlling the calcium homeostasis, the bone mineralization and remodeling and immune pathways. Additionally, VDR is responsible for the detoxification of both endogenous ligands and xenobiotics. The natural ligand of VDR is the active form of vitamin D called D3. It has been reported in several studies that the lack of Vitamin D leads to hypocalcemia and hypophosphatemia resulting in chronic kidney disease (CKD) also found in patients under chronic hemodialysis, mineral-bone disorders, osteoporosis and cancer (92, 94, 141, 142). In this review, we collected 3 articles that aimed at identifying novel VDR modulators for therapeutic purpose. 2 articles both used a combination of LB (pharmacophore‐based 3D‐QSAR models) and SB (docking and molecular dynamics) methods to identify respectively VDR inhibitors (93) and VDR agonists (94). Both models are associated with good correlation value (R²=0.8869 and R²=0.8676 respectively) and predictive score on the training set (Q²=0.8870 and Q²=0.8523 respectively). However, the low diversity and limited number of compounds used to train the model in the first study (93) and the difficulty to understand and thus reproduce the protocol used in the second one (94) may limit the applicability of these 2 models. In the third study (92), *de novo* design, docking and molecular dynamics was used to design new potential VDR agonists. However, except a redocking evaluation of D3, the performance of the protocol was not assessed retrospectively and no experimental test was conducted to validate the predicted hits. Additionally, one article (100) used data from the Tox21 initiative to build deep learning toxicological models for 35 NRs including VDR.

### 3.3 Monomeric orphan receptors

The members of this category of NR are characterized by an incomplete knowledge about their endogenous ligands and their ability to be activated as a monomer (or homodimer) by opposition to the orphan receptor that are partners to RXR (108). 3 NR studied during this review process and acting as monomers belong to this category (**Table 3**).

#### 3.3.1 Estrogen related receptors

ERR are orphan receptors, i.e. no endogenous ligands have yet been characterized for the ERR. They do not bind any natural hormones, not even estrogen despite their names (which has been chosen regarding the high sequence similarity in the DNA-binding domain between ERR and ER) but they do bind some synthetic estrogenic compounds (143). ERR are present in the human body under three main isoforms, ERRα, ERRβ and ERRγ that modulate cartilage development, mitochondrion organization and T-cell activation and differentiation. ERRα has particularly been investigated due to its tight similarity with ERα. Common DNA regions can be activated by both ERRα and ERα (144) and they shared common co-activators which may explain the fact that ERRα is involved in estrogen-related diseases. However, the lack of known endogenous ligand has impaired the establishment of protein-ligand interaction profile and the discovery of synthetic ligands. However, the Tox21 program has provide a huge amount of binding and activity data for ERR that could be used to build prediction models. Klimenko et al. (95), proposed a ligand-based (LB) approach to identify ERRα agonists by combining QSAR models. Each QSAR model is specific of a particular biological endpoint aiming at identifying potential ERRα agonists. The models were selected according to the balance between several statistical parameters i.e sensitivity, specificity, Accuracy, balanced accuracy, PPV and NPV. Other investigated ERRα ligands are inverse agonist and antagonist compounds that can be used for cancer patients with resistance to hormonal therapy. In order to identify such compounds, Chitrala et al. (96) used a library from KEGG COMPOUNDS, containing an ensemble of metabolic compounds, pharmaceutical and environmental compounds. This library was pre-filtered to select 8 compounds presenting a similarity score greater than 0.3 with an antagonist compound co-cristallized into the ERRα binding site. The ERRα structure in its antagonist conformation was then used to dock these compounds. Unfortunately, no biological evaluation of the proposed hit compound was achieved.

#### 3.3.2 Liver receptor homolog 1

LRH-1, also called NR5A2, plays an essential role in the well-functioning of the liver, the pancreas and the intestines by controlling the level of cholesterol, bile acids and pancreatic enzymes. LRH-1 is also involved in cell differentiation and associated with key developmental pathways. However, dysregulated LRH-1 activity and unexpected re-activation of the previously mentioned developmental pathways has been linked with breast, endometrial, intestinal and pancreatic malignancies (97). Limited data is available for LRH-1. In particular, LRH-1 is still classified as an orphan receptor, but several studies pointed out phospholipid species as possible endogenous ligands. This has impacted the development of LRH-1 modulators and of *in silico* models dedicated to LRH-1. Only one article dedicated to LRH-1 was found during the review process (97). This article published in 2013 presents a prospective structure-based screening with the aim of identifying the first synthetic LRH-1 antagonist compound. Using a docking method, 2 new LRH-1 antagonists were discovered and proposed to be used as a probe to help decipher LRH-1 mechanism of action.

#### 3.3.3 Retinoic acid-related orphan receptor

Three subtypes of ROR exist, namely RORα, RORβ and RORγ with its two isoforms, RORγ1 and RORγt (RORγ2). Each subtype presents a different pattern of expression, RORα being highly expressed in the brain, RORβ in the central nervous system, RORγ1 in the liver, the adipose tissue, the kidney, the small intestines, and the skeletal muscles and RORγt being exclusively expressed in cells of the immune system (145, 146). RORs are implicated in several key physiological functions. In particular, RORγt plays a major role in the differentiation of T-helper 17 (TH17) cells that produce the cytokine IL-17, itself involved in several diseases such as psoriasis, multiple sclerosis, rheumatoid arthritis and type 1 diabetes (98, 99). Additionally, the inhibition of RORγt stimulates the AR gene transcription and can be a strategy to follow for prostate cancer treatment (99). Identifying RORγt modulators emerges as a promising therapeutic strategy for all these conditions. However, until very recently, the known RORγt modulators were all sharing the same scaffold and efforts have been made towards the discovery of new RORγt modulators with original scaffolds. Two articles (98, 99) described SB *in silico* approaches to identify potent inverse agonists of RORγt that can be used in auto-immune diseases treatment. In the first article, docking and negative image-based methods (NIB) were both first evaluated retrospectively with RORγt experimental data extracted from the ChEMBL database to define docking score threshold and cutoff similarity value, respectively. These 2 methods were then used in parallel to screen a collection of more than 100000 molecules commercially available. Experimental tests validated as RORγt inverse agonists 11 up to the 34 predicted consensus hit compounds with an original scaffold.

### 3.4 Projects targeting several NR

Besides articles that were solely dedicated to a specific NR presented above, other projects focused on ensemble of NRs to provide more global NRs ligand identification models. Except one article (106), all these projects fall under a toxicological scope with the aim of identifying potential EDCs and understanding their mechanism of action. The most targeted NR combination is ER and AR due to extensive literature and data available. For example, Li et al. (31) studied the ability of some EDCs to present a dual activity by interacting with both AR and ER. To do so, they constructed ER binding models using a QSAR approach and these models were used to screen a database of AR antagonist compounds. It is to note that some of these projects are made available as webservers and software (12, 103–105). Open VirtualToxLab (105) is a software that estimates the toxicological potential of the screened compounds by relying on an automated combination of flexible docking and free energy calculations. Open VirtualToxLab focuses on 16 biological targets among which are 9 NR: AR, ERα, ERβ, GR, LXR, MR, PPARγ, PR, TRα, TRβ. The proof of concept of this software was established with a series of 2564 compounds yielding accurate predictions [C.f. **Table 2** of (105)]. Endocrine disruptome (104) is a freely available open-source project that allows users to test the ED potential of their compounds against 14 NR involved in several biological processes: AR, ER α, ER β, GR, PPARα, PPARβ, PPARγ, PR, RXRα, and TRα and TRβ. This webserver is based on docking models elaborated for each NR individually. The major drawback of this tool is the use of DUD-E decoys to validate the protocol. These decoys are putative inactive compounds, and not experimentally validated inactive compounds, which may bias the evaluation of the performance of the models. ProTox-II (103) is a webserver predicting the potential toxicity of small molecules by using 33 models of different toxicity endpoints. Among these endpoints are AhR, AR, ER and PPARγ signaling pathways. The models for each one of these NRs performed with high accuracy on the Tox21 dataset [C.f **Table S2** of (103)].

### 3.5 Related receptors: Aryl hydrocarbon receptors

Although AhR does not belong to the NR superfamily, this receptor displays functional and structural similarities with the members of this family, particularly the presence of a DBD and a LBD activated upon ligand binding (147). During the review process, we noticed that AhR is often associated with other NR in toxicological studies and in the EDCs context and we decided to list also the *in silico* studies dedicated to this receptor (**Table S1**). AhR is expressed in various tissues such as the lung, liver, kidney, skin, sleen and placenta. It can be activated by several hazardous chemicals such as polycyclic aromatic hydrocarbons (PAHs) and persistent organic pollutants (POPs) especially dioxins and polychlorinated biphenyls (PCBs). These chemicals result from combustion and are massively found in the air (148). Due to its pivotal role as an environmental pollution sensor and mediator, AhR emerges as a major target for toxic compounds which is illustrated by the predominance of reported toxicological projects (8 toxicological among 10 in total). Most of the data used in these projects are collected from the literature and these studies are in their large majority dedicated to specific chemical series of environmental compounds that have been proved experimentally to be able to interact with AhR (71, 149, 150). The corresponding models are associated with high predictive power, but their applicability domain is relatively limited. Additionally, data from the Tox21 project was also used with both SB and LB methods. However, the application of SB methods is limited by the fact that the 3D structure of this receptor has not been solved yet. A preliminary homology modelling step is thus necessary in all the studies relying on SB methods to obtain a model of the AhR LBD. AhR belongs to the PAS-domain protein group (151) and usually the structure of the PAS-B of the Hypoxia Inducible Factor 2 α (HIF-2α) is used (152, 153) sharing 31% of sequence similarity and 52% identity with the target considered as the highest sequence identity and similarity. More recently, potential therapeutic applications of AhR modulation have emerged. AhR activation has been shown to be involved in hematopoiesis and inflammation process especially the production of inflammatory cytokines whereas AhR repression has been shown to be beneficial in anti-cancer therapies especially for glioblastomas and breast cancers (154). Two therapeutic projects were analyzed in this review, the first being a LB model aiming to discover new AhR antagonists. For this purpose, Parks et al. used successively the Rapid Overlay of Chemical Structures (ROCS) and the electro-static overlap to identify one compound that yield promising experimental results in three *in vitro* and *in vivo* AhR-dependent assays (154). The second therapeutic project applied a SB protocol to decipher the molecular mechanisms behind the AhR activation or inhibition (152). In this article, several entries for the template protein were used to simulate the flexibility and plasticity of the binding domain and homology models were selected and used to perform the docking of 10 representative AhR agonists from different chemical classes. The outcome of docking coupled with MD simulations allowed the analysis of the predominant poses and thus the description of ligand binding and the identification of the interacting residues within AhR.

## 4 Studied methods and associated data

### 4.1 Computational methods

**Figure 3:**
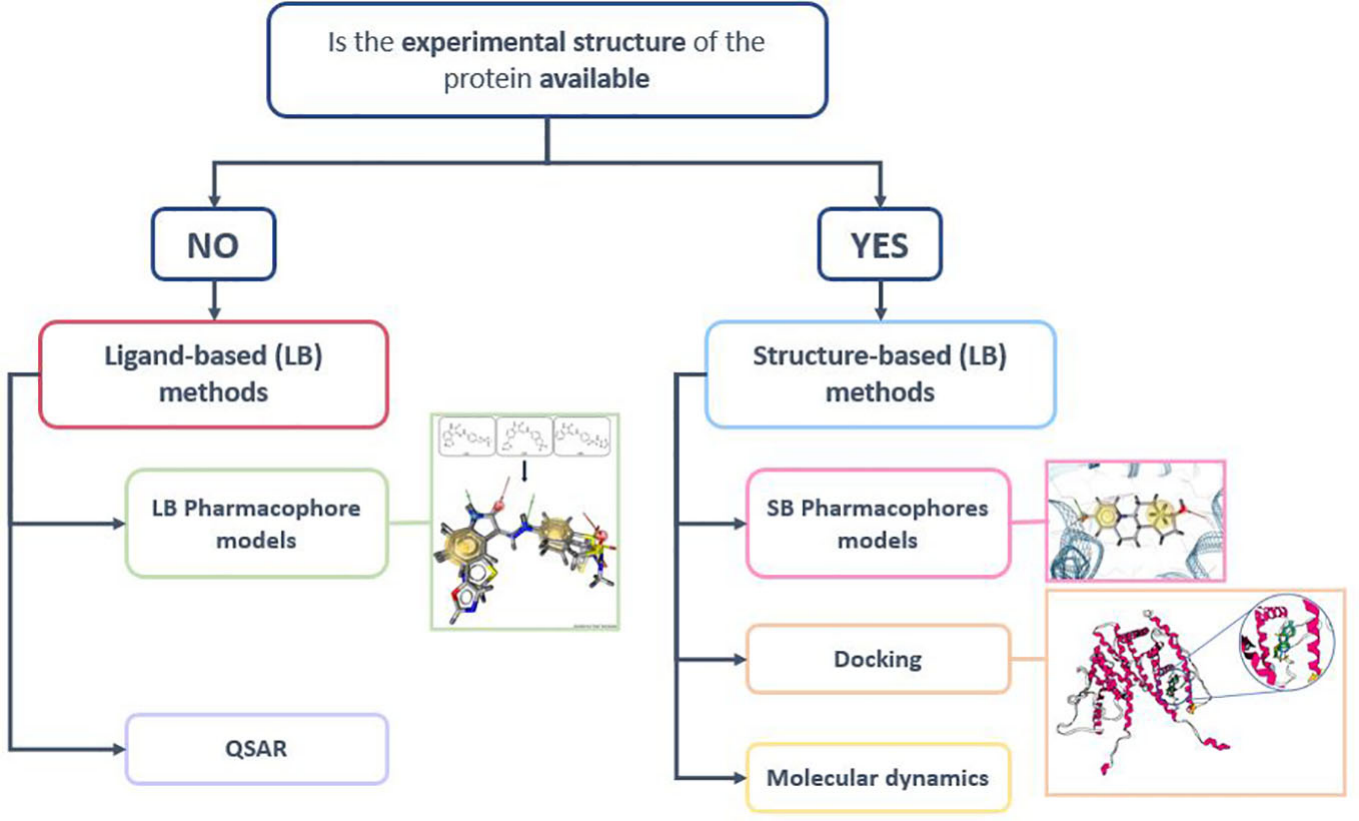
Structure-based (SB) and Ligand-based (LB) screening methods (155).

Along this review process, we analyzed models dedicated to the identification of NRs ligands obtained with a very large spectrum of *in silico* methods categorized in two approaches i.e SB and LB as shown in **Figure 3**.

#### 4.1.1 Molecular dynamics simulations

MD simulations are used to study the dynamic of a biological system as a function of time. The movements of the system are computed through the integration of Newton’s equation of motion. MD simulations can thus be used to consider the conformational changes associated with the NRs LBD. Indeed, the NRs LBD is characterized by its hydrophobicity and flexibility allowing binding of ligands of different sizes and shapes. NRs structures are subject to various modifications occurring after several events such as the DNA transcription or the binding of the ligand (15). The helix H12 is known to modify its orientation according to the bound-ligand profile. MD can thus be used to span the NRs flexibility and deliver unbiased theoretical structures, but also to assert the stability of a ligand-protein complex and study the time of residence of the ligand. In the reviewed articles, MD has been used for several projects relevant to AR, ER, LXR, PPAR, TR and VDR always in complement of another computational method such as QSAR (74, 150) or pharmacophore models (93, 94) and frequently prior or after docking simulations (66, 75, 152).

#### 4.1.2 Docking

Protein-ligand docking is a SB method used to predict the ability of a compound to interact with a target. Docking methods used a search algorithm that generate multiple potential conformations of the molecular complex and a scoring function to rank them. Docking can be used to propose structural hypotheses on the dominant binding mode of a compound, to screen large libraries of compounds and to rank them according to docking scores. In this way, docking is used to prioritize and reduce the number of compounds that should be experimentally tested (156). Docking methods can be applied to any target for which an experimental 3D structure is available. Consequently, docking methods were largely employed to develop predictive models of NR-ligand interactions and for each of the 14 reviewed NR subfamily, at least one project using a docking method was available. However, the docking protocol used must be finely tuned for each NR. A first important criterion is the rational selection of the initial docking structure(s) since the NRs LBDs undergo significant conformational changes upon ligand binding, dependent of the pharmacological profile of the ligand. NRs agonist compounds tend to stabilize the interactions between the residues of helix H12 and other helices (H3, H5 and H11) of the LBD, creating a lid shape of the H12 over the ligand. In contrast, other compounds (antagonists or partial agonists) are not able to stabilize the previous interactions and the observed repositioning of the H12 is different. The choice of an agonist-bound or antagonist-bound structure may thus impact the docking performances in predicting NRs ligands binding (15). This is particularly important for docking protocols aiming to discover therapeutic compounds targeting the NRs as a specific pharmacological profile is usually targeted and the corresponding agonist- or antagonist-bound structure should be selected. In contrast, EDCs can present different pharmacological profiles and it is thus important to evaluate both type of structures to ensure that the screening is able to retrieve all EDCs regardless of their pharmacological profile. In the reviewed articles, two main docking approaches have been listed: single and an ensemble structure docking. In the ensemble structure docking approach, a ligand is successively docked against several protein conformations (Multiple PDB entries or snapshots extracted from MD simulations or a gaussian transformation of the atom localization) and the results are post processed to only keep the best score among all the structures. This approach provides a better picture of the protein flexibility but is more computationally and time expensive and it is not always associated to enhanced docking performances (157).

#### 4.1.3 Pharmacophore modeling

A pharmacophore model is defined by the IUPAC as “an ensemble of steric and electronic features that is necessary to ensure the optimal supramolecular interactions with a specific biological target and to trigger (or block) its biological response” (158). Two types of pharmacophore models exist (1): LB pharmacophore models established upon the superimposition of known active molecules and the retrieval of the common chemical features that are necessary for the bioactivity (2); SB pharmacophore models based on the structure of a protein or more usually of a complex protein-ligand by probing the possible interactions in the macromolecule binding site (159).

In total, 17 reviewed articles used pharmacophore modelling. The generated pharmacophore models are in a large majority combined with other computational models (QSAR and docking models) and only two publications present a protocol combining SB and LB pharmacophores (76, 79). It is to note that both LB and SB pharmacophores taken individually have a predictive power limited by the data used to train the model, defining their applicability domain. The protocol combining pharmacophore models with other *in silico* methods are described in the reviewed articles and may help overcoming this limit.

#### 4.1.4 QSAR models

QSAR (Quantitative Structure Activity Relationship) is a LB method relying on statistical models to predict the biological activity of compounds. Since their introduction, QSAR models have known several evolutions from simplistic linear and classification models to the use of more elaborated algorithms like artificial neural networks (ANN) and deep learning (DL). In total, 48 of the reviewed articles used QSAR models with the majority (19) adopting classical 2D machine learning methods like Random Forest or SVM. The remaining studies present models obtained using ANN (4), DNN (3) or 3D QSAR methods such as CoMFA and CoMSIA (7). The classical QSAR models rely on a set of descriptors that encode chemical structures, describe physiochemical properties and that can be obtained from quantum chemical calculations (14, 88). Because a huge number of predefined descriptors can be generated, it is important to correctly select the ones that will accurately translate the link between the chemical structure and the associated activity (160). A classical approach is illustrated in the publication of Wang et al. (88) in which QSAR models dedicated to the identification of agonist and antagonist ligands of TR are presented. In this study, the software Dragon was used to initially generate over 1600 descriptors. This number was then reduced by discarding the descriptors highly correlated and selecting only those directly related to the structure, intuitive and easy to understand. Conversely, DL methods emancipate from descriptors computation and selection. Instead, DL methods ensures automatic feature extractions during the training phase. DL methods are thus particularly adapted for areas where existing predefined descriptors have not been crafted yet like macrocycles or the modeling of therapeutic peptides (161). However, DL methods also present some drawbacks, the first being the so-called “black box effect”. DL methods compared to more simplistic algorithms are not easily understandable and deciphering the molecular mechanisms associated with the prediction may be a tough task. Moreover, DL requires higher computing resources which can be a limiting factor.

Similarly to the pharmacophore models, QSAR models’ predictions are only accurate for compounds that are similar to the ones used to train them (21, 45, 162). An applicability domain (AD) can thus be defined with descriptors used to build the QSAR model (21, 25).. Implementing the AD has proven to enhance the confidence in the models by reducing the number of false negatives (25). However, prediction will only be achieved for compounds that are within the limit of the AD, i.e. within a defined descriptors similarity threshold with their nearest neighbors of the training set (45). In order to enhance the chemical space for which prediction can be made, QSAR models can be combined with other ones with complementary ADs or with a SB model such as docking (67, 68, 74, 81).

#### 4.1.5 Data splitting

An important step prior the generation of LB models is to split the available ligands data into train and test set (160). The stake of a good data split is to obtain a test set that is somehow representative of the training set chemical space. In most of the reviewed article, data was split randomly as it is commonly done to avoid biased evaluation of the model. However, this randomness may lead to an uneven distribution of active and inactive compounds between the train and the test set especially if the active/inactive ratio is initially low. In this sense, Capuzzi et al. (101) build QSAR models relying on two sets of databases: (1) the native Tox21 set of data with a ratio of active: inactive equal to 1:10 and (2) a balanced set generated down-sampled from the inactive data of the Tox21 native dataset i.e. for each active compounds an equivalent inactive was selected either randomly or based on the highest Tanimoto score within the inactive pool of compounds. It is to note that using the balanced set leads to a decrease in the accuracy of the model in comparison to when they used the native (unbalanced) dataset. Randomly splitting data may also result in a test set that is not representative of the train especially when the initial data are structurally diverse. Wang, Xing et al. (88) used self-organizing map (an ANN algorithm) to project an ensemble of structurally diverse compounds on a low dimensionality grid. This helped visualizing the space and the selection of a test set representative of the overall dataset and lead to average accuracies of 83.1–97.2%. In other studies, the trade-off between randomness and unbiased subdivision was achieved with bootstrapping. Several splits were done, and models were built an evaluated with the associated train/test output. The split with the best results was kept for the following model optimization (29, 163).

#### 4.1.6 Combinations of methods

It is to note that each computational method has limitations. For example, QSAR methods are not suited on their own to be used for HTS. They are more efficient at generating focused data and requires exhaustive model training and validation steps. Moreover, QSAR and pharmacophores modelling share the common particularity of being only efficient on molecules falling within a domain of applicability (162). A crucial point in the docking workflow is the choice of the scoring function as no universal scoring function exists yet (164). Each individual method can be at the origin of new structural information that can be combined to enable a better understanding of the mechanism of action. A solution to overcome each method limitations and take advantage of their complementarity is to use a combination of methods (28, 165, 166). Computational methods can be combined in integrative approaches using hierarchical or consensus screening (27–29, 48, 60, 100, 163) (for further references c.f. **Table 1**–**4**).

### 4.2 Databases

A chemical database (DB) is an organized collection of compounds with relevant information on their chemical structures together with activity data collected from *in vivo* and/or *in vitro* experiments and sometimes *in silico* predicted activities. Several DB dedicated to NR exist and we decided in this review, to focus on the ones available in open access as presented in **Table 5**.

**Table 5:** Example of databases including or dedicated to nuclear receptors.

Database	Link	Composition	Specific to NR only
Binding DB	https://www.bindingdb.org/bind/index.jsp	As of November 8, 2021, BindingDB contains 2,369,418 binding data for 8,634 protein targets and 1,023,385 small molecules	No
ChEMBL	https://www.ebi.ac.uk/chembl/	manually curated database: 2.1 M compounds	No
Drugbank	https://go.drugbank.com/	14,585 drugs and several targets like enzyme, transporters and carriers	No
ZINC database	https://zinc.docking.org/	contains over 230 million purchasable compounds in ready-to-dock, 3D formats.	No
Tox21	https://tripod.nih.gov/tox21	The list of ToxCast and Tox21 chemicals suspected to be a hazard for human and environmental health and associated information for 9,403 unique substances.	No
ToxCast	https://www.epa.gov/chemical-research/toxcast-chemicals		No
DUD-E	http://dude.docking.org/	22,886 active compounds and their affinities against 102 targets, an average of 224 ligands per target and 50 decoys for each active having similar physico-chemical properties but dissimilar 2-D topology.	No
DSSTox	https://comptox.epa.gov/dashboard/chemical-lists/tox21sl	launched in 2004, currently exceeds 875K substances spanning hundreds of lists of interest.	No
EDKB	https://www.fda.gov/science-research/endocrine-disruptor-knowledge-base/accessing-edkb-database	Data for more than 3200 chemicals	Yes (ER and AR)
EABD	https://www.fda.gov/science-research/bioinformatics-tools/estrogenic-activity-database-eadb	18,114 estrogenic-activity data points collected for 8,212 chemicals tested in 1,284 binding assays, reporter-gene assays, cell-proliferation assays, and in-vivo assays in 11 different species.	Yes (ER)
NR-DBIND	http://nr-dbind.drugdesign.fr/	15,116 positive and negative interactions data are provided for 28 NRs together with 593 PDB structures	Yes
NR-List BDB	http://nrlist.drugdesign.fr/	9,905 compounds and 339 structures of the NRLiSt BDB	Yes
ONRLDB	https://academic.oup.com/database/article/doi/10.1093/database/bav112/2433243	∼11 000 ligands, of which ∼6500 are unique.	Yes
NURA	https://www-sciencedirect-com.proxybib-pp.cnam.fr/science/article/pii/S0041008X20303707?via%3Dihub	bioactivity data for 15,247 molecules and 11 NRs	Yes (ERα andβ, PPARGαγ, and δ, AR, GR,PR, FXR,RXR and PXR)

*In silico* studies focusing on NR are numerous and the choice of the databases to use to construct predictive models depends on the aim of the project. Moreover, and especially for drug design projects, some research teams chose to use in house databases i.e. a collection of compounds issued as a result of experimental work in the laboratory. These databases can be composed of one or several chemical series originating from a single hit or a known drug scaffold or, in some cases focused libraries. Finally, some projects rely on several existing databases, either to combine the molecules resulting in larger databases for the same purpose (therapeutic or toxicological) or use a database for the training step and another for the external validation. For example, Réau et al. (29) developed a docking and a pharmacophore modeling strategy for identifying AR agonist compounds relying on the NR-DBIND data and used as an external validation set the compounds from the tox21 challenge.

#### 4.2.1 Assay consideration

In the scientific literature, biological data available for NRs ligands represent various assays endpoints sometimes measured from different laboratories and companies. Indeed, two main assays were used in the reviewed studies to assess NRs ligands potency: binding assay and gene reporter assay (34). These assays attributed to each compound a quantitative value that may vary according to the laboratory in charge of the experiment. In the *in silico* modelling field, the experimental data are used to compute predictive models for molecules with no related experimental information. For some methods such as QSAR, the numerical value associated with the experimental test can directly be integrated to construct the models. For other method, such as docking and pharmacophoric modeling, these data usually need to be converted into a binary variable with 2 possible values: “active compounds” and “inactive compounds”. This is necessary to optimize and select the prediction models able to distinguish between both categories. Indeed, to evaluate the predictive power of a model, the number of correctly predicted active and inactive (TP and TN) as well as the number of wrongly predicted compounds (FP and FN) are used to compute metrics such as sensitivity, sensitivity and enrichment for example. The binarization of the data is a crucial but not straightforward step because a threshold must be defined to separate the data. Additionally, because various biological endpoints are considered, the concordance between the thresholds of activities defined for each assay should be assessed. Lunghini et al. (167) defined the “degree of agreement” parameter to pinpoint the differences between various assays used to label compounds that interact with ER and AR in the context of EDCs models. To do so, they performed multiple pairwise comparisons of various binding assays result among 4 data sources for ER binding compounds and 3 data sources for AR binding compounds. The degree of agreement was calculated as the average number of sources agreeing on a given label for each compound. Their analysis showed that there is a lack of concordance between experiments for 42% of the compounds. This study highlighted the danger of merging assays with different biological meanings (167) and considering the positive outputs as interacting compounds regardless of the mechanism of action. However, it is a commonly used protocol, often driven by the lack of appropriate data or the blurry definition of the mechanism of action. This is especially the case in the toxicological context of the study related to EDCs and in this review, we came across several studies performing the merging of data obtained from different assays and different sources. Some studies, aware of this issue, also developed various approaches to limit the associated bias. Sakkiah et al. (56) calculated the concordance between the train and the test set since originated respectively from binding and amplification assays. Manganelli et. al (28), compared the train and the test data to define concordance and exclude a part of the data that falls under a “dangerous” segment. Finally, Zhang et al. (128) normalized the activity of their test set to their training data when constructing their models.

#### 4.2.2 Data balance

Along with active compounds, it is also important to construct, optimize and evaluate NRs prediction models to include negative data (168). Positive data are usually resulting from biological or cellular *in vivo assays* but, negative data has long been constituted by presumed inactive compounds (121). Since negative data have a crucial impact in influencing the performance of a model (168), recent efforts have been made to include carefully selected and experimentally validated inactive compounds [NR-DBIND (169)]. An additional issue is the proportionality between active and inactive compounds in DB. When collecting data from scientific literature, very few inactive compounds are retrieved. Conversely, in databases presenting high throughput screening results, inactive compounds usually outnumbered the active ones. In this case, the active substances represent a low proportion out of all tested chemicals (95). A performant model is dependent on clean, diverse and accurate data (95, 165), and unbalanced data (either in favor of the active or the inactive counterpart) can impact prediction models building (55). Clustering methods can be used to select smaller subsets of representative active or inactive compounds. For example, Another solution is the use of a structural similarity filter based on fingerprints and the application of a similarity metric cutoff (for example Tanimoto) in order to select sparse compounds to scour the entire chemical space (170). In the case of unbalanced data sets, the classical metrics are not the best suited to evaluate the predictive power of a model. In this case some metrics like the MCC and the F1 score can help in assessing the ability of a model to correctly predict each category of compound.

### 4.3 Level of reproducibility

Reproductible science is a quality standard that the scientific community values as it helps producing high quality, reliable and efficient research project (171). It has been proven that reproducibility together with methods, reporting, dissemination, evaluation and incentives are key elements for the scientific process (172). However, it has also been asserted that the field of preclinical research suffers from the inability to replicate findings published in high-profile journals (171). Although it is difficult to exactly replicate results in biology systems due to their inherent variability, some recommendations on good practice could be listed to alleviate the trustworthiness of the scientific work and the validity of the major conclusions (171). Reinforcing the policies on data and code sharing is one of these recommendations (171, 173).

Through this review, we decided to evaluate this point in the NRs *in silico* research field. For each reviewed model, the level of reproducibility was graded according to the availability of databases and the model procedure’s parameters and/or code. The “high” grade was used to describe models providing both components within the article, the “medium” was associated with articles with partial available data. Finally, the reproducibility was described as “low” for models where no data were available. For most of the reviewed articles’ reproducibility was rated “medium” (52 out of 89 articles) and only a low proportion was described to have a “high” reproducibility (16 out of 89). These results focused on the NRs are in concordance with other large-scale studies (173). For example, in a study of 2011, the analysis of 500 papers published in the top 50 journals across scientific fields showed that only 9% of these papers enclosed full primary data (171). The reinforcement of collaborations between research teams following the example of COMPARA (20) and CERAPP (36) consortiums or the Tox21 challenge (174) can improve the reproducibility since it creates accountability in exchange of a benchmarked data (172). However, according to our classification, no particular tendency was observed over the 10 year covered by this review neither towards increase of the number of articles labeled with “high” reproducibility nor towards decrease of the number of articles associated with “low” reproducibility.

## 5 Conclusion

Nuclear receptors are a large family of transcription factors involved in several biological process and their impairment result in several pathologies. Moreover, this protein family can be targeted by toxic compounds leading to the disruption of the normal functioning of NRs and especially the disruption of the endocrine system by a family of compounds called endocrine disrupting chemicals or EDCs. Being in the center of both therapeutic and toxicological concerns, NRs are widely studied to find new cures but also to unravel the potential toxicity of environmental compounds such as pesticides, cosmetics or additives. In the last decades and with the emergence of bioinformatics and virtual screening techniques, computational models dedicated to NR were developed. The computational capabilities were put to use to interpretate and analyze the experimental data and build predictive models to unravel new drug hit and forecast potential toxicants. This article is a review of the studies dedicated to NR ligands prediction for both therapeutic and toxicological purposes, published in the last decade. 89 articles concerning 14 NR subfamilies were carefully read and analyzed in order to retrieve the most commonly used computational methods to develop predictive models, to retrieve the databases deployed in the model building process. Some issues facing the model building process were addressed like the assays endpoint discrepancies and data balance were also addressed and how authors managed to overcome them. This review emerged from the need to identify most of the *in silico* initiatives undertaken on NR and can be used as a starting point for future investigations on the subject not only to appreciate the importance of NR in both therapeutic and toxicological fields, but also to learn from previous experiences, encourage the elaboration of more accurate predictions and motivate collaborations.

## Author contributions

Conceptualization: AS, MR, and NL. Methodology: AS, MR, and NL. Validation: NL, MR, and MM. Formal analysis: AS and NL. Investigation: AS. Resources: AS, MR, NL. Data collection and curation: AS. Writing—original draft preparation: AS. Writing—review and editing: AS, MR, NL, and MM. Supervision: NL. Project administration: MM. Funding acquisition: AS, NL, and MM. All authors contributed to the article and approved the submitted version.

## Funding

AS is recipient of a MESRI (Ministère de l’Enseignement supérieur, de la Recherche et de l’Innovation) fellowship.

## Conflict of interest

The authors declare that the research was conducted in the absence of any commercial or financial relationships that could be construed as a potential conflict of interest.

## Publisher’s note

All claims expressed in this article are solely those of the authors and do not necessarily represent those of their affiliated organizations, or those of the publisher, the editors and the reviewers. Any product that may be evaluated in this article, or claim that may be made by its manufacturer, is not guaranteed or endorsed by the publisher.
