# Supplementary material for: Review of in silico studies dedicated to the nuclear receptor family: Therapeutic prospects and toxicological concerns
Source: Front Endocrinol (Lausanne). 2022 Sep 13;13:986016. doi: 10.3389/fendo.2022.986016 (PMC9513233; doi:10.3389/fendo.2022.986016)
Supplement: Supplementary file 1 [file Table_1.docx]

Table S 1 : Review of the different initiatives dedicated to AhR

| **Receptor** | **methods** | **Approach** | **Database** | **Prospective or Retrospective** | **Reproducibility** | **Application** | **Year** | **Ref** |
| --- | --- | --- | --- | --- | --- | --- | --- | --- |
| AhR | Docking and 3D-QSAR | both | Collected from the literature (65 compounds including 24 PCDDs, 27 PCDFs, and 14 PCB) | retrospective | Low | Toxicological | 2011 | (1) |
| AhR | Docking | SB | Collected from the literature (5 compounds: THS-017, THS-020, TCDD and 2 co-crystallized ligands) | retrospective | Medium | Toxicological | 2011 | (2) |
| AhR | QSAR (Partial least square analysis) | LB | Collected from the literature (18 PBDE compounds) | retrospective | High | Toxicological | 2012 | (3) |
| AhR | docking, MD and 3D-QSAR | both | Collected from the literature (50 HO-PCBs compounds) | retrospective | Low | Toxicological | 2013 | (4) |
| AhR | docking based 3D-QSAR (CoMFA) | LB | Collected from the literature (78 compounds) | retrospective | Medium | Toxicological | 2013 | (5) |
| AhR | Rapid Overlay of Chemical Structures (ROCS), electro-static overlap | LB | 445,418 compounds from ChemBridge Corporation (San Diego, CA) and 731,288 compounds from Enamine | prospective | Medium | Therapeutic | 2014 | (6) |
| AhR | docking | SB | Collected from the literature (17 PAHs compounds) | retrospective | Low | Toxicological | 2015 | (7) |
| AhR | Docking and MD | SB | Collected from the literature (10 compounds) | retrospective | Medium | Therapeutic | 2019 | (8) |
| AhR | Docking | SB | Collected from the literature (18 PBDEs compounds) | retrospective | Low | Toxicological | 2020 | (9) |
| AhR | QSAR:DNN | LB | Commercial library (201 compounds) | retrospective | Medium | Toxicological | 2020 | (10) |

**References**

1. Li F, Li X, Liu X, Zhang L, You L, Zhao J, et al. Docking and 3D-QSAR studies on the Ah receptor binding affinities of polychlorinated biphenyls (PCBs), dibenzo-p-dioxins (PCDDs) and dibenzofurans (PCDFs). Environ Toxicol Pharmacol. 2011 Nov;32(3):478–85.

2.: I. Motto, An. Bordogna, A. A. Soshilov, M. S. Denison and Laura Bonati. New Aryl Hydrocarbon Receptor Homology Model Targeted To Improve Docking Reliability | Journal of Chemical Information and Modeling. [cited 2022 Sep 2]; Available from: https://pubs-acs-org.proxybib-pp.cnam.fr/doi/full/10.1021/ci2001617

3. Gu C, Goodarzi M, Yang X, Bian Y, Sun C, Jiang X. Predictive insight into the relationship between AhR binding property and toxicity of polybrominated diphenyl ethers by PLS-derived QSAR. Toxicol Lett. 2012 Feb 5;208(3):269–74.

4. Cao F, Li X, Ye L, Xie Y, Wang X, Shi W, et al. Molecular docking, molecular dynamics simulation, and structure-based 3D-QSAR studies on the aryl hydrocarbon receptor agonistic activity of hydroxylated polychlorinated biphenyls. Environ Toxicol Pharmacol. 2013 Sep;36(2):626–35.

5. Yuan J, Pu Y, Yin L. Docking-based three-dimensional quantitative structure-activity relationship (3D-QSAR) predicts binding affinities to aryl hydrocarbon receptor for polychlorinated dibenzodioxins, dibenzofurans, and biphenyls. Environ Toxicol Chem. 2013 Jul;32(7):1453–8.

6. Parks AJ, Pollastri MP, Hahn ME, Stanford EA, Novikov O, Franks DG, et al. In Silico Identification of an Aryl Hydrocarbon Receptor Antagonist with Biological Activity In Vitro and In Vivo. Mol Pharmacol [Internet]. 2014 Nov 1 [cited 2022 Jan 13];86(5):593–608. Available from: https://molpharm.aspetjournals.org/content/86/5/593

7. Lee S, Shin WH, Hong S, Kang H, Jung D, Yim UH, et al. Measured and predicted affinities of binding and relative potencies to activate the AhR of PAHs and their alkylated analogues. Chemosphere. 2015 Nov;139:23–9.

8. Giani Tagliabue S, Faber SC, Motta S, Denison MS, Bonati L. Modeling the binding of diverse ligands within the Ah receptor ligand binding domain. Sci Rep [Internet]. 2019 Jul 23 [cited 2021 Sep 9];9:10693. Available from: https://www.ncbi.nlm.nih.gov/pmc/articles/PMC6650409/

9. Gu C, Cai J, Fan X, Bian Y, Yang X, Xia Q, et al. Theoretical investigation of AhR binding property with relevant structural requirements for AhR-mediated toxicity of polybrominated diphenyl ethers. Chemosphere. 2020 Jun;249:126554.

10. Matsuzaka Y, Hosaka T, Ogaito A, Yoshinari K, Uesawa Y. Prediction Model of Aryl Hydrocarbon Receptor Activation by a Novel QSAR Approach, DeepSnap-Deep Learning. Molecules. 2020 Mar 13;25(6):E1317.
